# Internet-Based Healthcare Knowledge Service for Improvement of Chinese Medicine Healthcare Service Quality

**DOI:** 10.3390/healthcare11152170

**Published:** 2023-07-31

**Authors:** Xiaoyu Wang, Yi Xie, Xuejie Yang, Dongxiao Gu

**Affiliations:** 1 The Department of Pharmacy, Anhui University of Traditional Chinese Medicine, Hefei 230031, China; xywang0551@ahtcm.edu.cn; 2 The School of Management, Hefei University of Technology, Hefei 230009, China; xuejie_y@mail.hfut.edu.cn (X.Y.); dongxiaogu@yeah.net (D.G.)

**Keywords:** quality of healthcare service, internet-based health service, Chinese medicine, healthcare knowledge service

## Abstract

With the development of new-generation information technology and increasing health needs, the requirements for Chinese medicine (CM) services have shifted toward the 5P medical mode, which emphasizes preventive, predictive, personalized, participatory, and precision medicine. This implies that CM knowledge services need to be smarter and more sophisticated. This study adopted a bibliometric approach to investigate the current state of development of CM knowledge services, and points out that accurate knowledge service is an inevitable requirement for the modernization of CM. We summarized the concept of smart CM knowledge services and highlighted its main features, including medical homogeneity, knowledge service intelligence, integration of education and research, and precision medicine. Additionally, we explored the intelligent service method of traditional Chinese medicine under the 5P medical mode to support CM automatic knowledge organization and safe sharing, human–machine collaborative knowledge discovery and personalized dynamic knowledge recommendation. Finally, we summarized the innovative modes of CM knowledge services. Our research will guide the quality assurance and innovative development of the traditional Chinese medicine knowledge service model in the era of digital intelligence.

## 1. Introduction

The acceleration of industrialization, urbanization, and population aging has made health the most pressing issue in the international community. The World Health Organization’s 13th General Programme of Work has identified the improvement in population health as its third strategic priority, with the aim of having one billion more people enjoying enhanced health and well-being by the end of 2025 [1]. In the context of today’s rapid social development, there has been a notable and ongoing increase in public health awareness. Specifically, people have shifted away from seeking treatment for isolated ailments, and have instead prioritized a more comprehensive approach to health management that spans their entire lifecycle. This approach is characterized by a focus on personalized care that addresses a broad range of factors influencing an individual’s health and well-being [2]. The Healthy China 2030 Planning Outline proposes integrating the advantages of Chinese medicine (CM) with health management to provide people with comprehensive, lifelong health services [3]. CM does not have an accurate definition at present, and it is generally considered to be a medical theory system gradually formed and developed through long-term medical practice under the guidance of Chinese cultural thought. Traditional Chinese medicine (TCM) posits that illnesses stem from an imbalance in a person’s life-force energy, known as ”Qi”, and its objective is to reestablish harmony within the individual [4]. Modern CM is a combination of TCM and modern medical technology. CM services have a wide range of applications, making them suitable for the treatment, rehabilitation, and health management of various types of diseases, particularly chronic and geriatric conditions. Numerous studies have confirmed the therapeutic and ameliorative effects of CM on chronic diseases, including cancer [5,6,7], diabetes [8], cardiovascular diseases [9,10], and neurological disorders [11,12] such as epilepsy, Alzheimer’s disease, Parkinson’s disease, depression, and cerebral ischemia. Additionally, CM has been shown to be effective in treating skin diseases [13,14], infertility [15], and other ailments. It is a reliable and valuable healthcare option for individuals seeking complementary or alternative therapies. Furthermore, CM has demonstrated its critical role in responding to major epidemics, including SARS and the novel coronavirus (COVID-19) [16,17,18]. The Statistical Bulletin on the Development of China’s Health Care in 2021, released by the National Health Commission of China in July 2022, showed that the total number of medical consultations at CM medical and health institutions nationwide reached 1.2 billion in 2021, representing a 13.7% increase from the previous year [19]. The survey results released by the National Administration of Traditional Chinese Medicine in China in 2021 showed that the health literacy of CM culture has been increasing yearly within five dimensions: basic concepts, healthy lifestyle, suitable methods for the public, cultural knowledge, and the ability to understand information. The percent of the population with health literacy in CM reached 20.69% in 2020 [20]. These figures indicate that increasingly more CM health services are being accepted and popularized by the public. However, despite the vast market and globalization of CM services, the safety and efficacy of CM have been subject to questioning by both domestic and international academic communities and the public because of significant differences between CM and modern medicine in terms of evidence-based practice and quality control. Furthermore, China’s CM service industry still faces challenges such as low-quality primary CM services, difficulty in allocating high-quality medical resources at grassroots levels, and obstacles in implementing the tiered medical diagnosis and treatment system.

In recent years, the rapid development of new-generation information technologies such as mobile internet, big data, 5G communication technology, cloud computing, artificial intelligence, and the internet of things has facilitated the intelligentization of society, and the healthcare industry is undergoing continuous transformation and innovation. The concept of precision medicine has been proposed to fulfill the growing demand for better health and quality of life. The medical mode has shifted toward the 5P mode, which consists of preventive, predictive, personalized, participatory, and precision medicine. Academia has extensively and deeply researched the combination of new-generation information technologies with healthcare. Liu et al. conducted research on chronic disease management through deep learning by studying data from video-sharing social media platforms [21]. Bobroske et al. studied the effects of early postoperative intervention on patients’ long-term use of opioid drugs by constructing a model of patients using opioid drugs [22]. Hajjar and Alagoz developed a randomized modeling framework that provides an accurate solution algorithm and personalized disease screening decision for chronic disease patients or potential chronic disease patients [23]. In addition, multiple scientific studies have shown the significant role of information technology in promoting health and well-being during the COVID-19 pandemic [24,25,26,27].

The application of new-generation information technology in the healthcare industry has also created opportunities for the transformation and upgrading of CM services. The Opinions on Promoting the Inheritance, Innovation and Development of Chinese Medicine, issued by the Central Committee of the Communist Party of China and the State Council in 2019, specifically emphasized the full use of new-generation information technologies such as big data and artificial intelligence in CM services, and promoted the deep integration and development of new-generation information technologies with CM health services. Compared with other disciplines, CM is an empirical science that relies more heavily on experience, and its knowledge is more complex and ambiguous. Currently, scholars and experts have achieved certain results in research on the internationalization, standardization, security, and application of artificial intelligence technologies such as deep learning and case-based reasoning (CBR) in CM [28,29,30]. However, there are still many gaps in the governance of cross-organizational, multimode, and heterogeneous data, organization of CM case knowledge, dynamic updating of knowledge, human–machine collaboration in CM knowledge discovery, and full-cycle personalized proactive knowledge services, which limit the accuracy and capabilities of CM intelligent knowledge services.

In response to the 5P mode transformation and upgrading of the demand for CM services, scholars have started to focus on research into smart CM knowledge services relying on the new generation of information technology. Therefore, this paper summarizes the concept and main features of smart CM knowledge services, conducts an academic review of the current research in this area, analyzes the smart CM knowledge service mode, and explores innovative management methods for smart CM knowledge services. This has significant implications for promoting the safe, effective, and reasonable clinical application of CM, leveraging its unique advantages and benefiting people’s health.

## 2. Concept and Characteristics of Smart CM Knowledge Services

Medical services are crucial to people’s lives, health, and safety, and rely heavily on experience and knowledge, with high demands for service accuracy. Relying solely on data or intuition-based solutions can often lead to significant risks. Therefore, the medical and health fields require additional support from domain-specific knowledge.

### 2.1. The Connotation of Smart CM Knowledge Services

In the field of CM, three types of knowledge are commonly encountered: The first type is general medical knowledge of CM, which includes its basic concepts, principles, and laws, as well as knowledge of clinical diagnosis and treatment. CM clinical diagnosis and treatment knowledge mainly covers the philosophical foundation of CM, as well as the basic theories of its understanding of human physiology, diseases, and their prevention and treatment. The second type consists of medical and health case knowledge that contains rich expert knowledge, and the third type is medical and health reasoning knowledge obtained through various intelligent algorithms. To effectively address complex medical and health management decision-making problems with high risk, it is necessary to integrate these three types of knowledge: general medical knowledge, medical and health case knowledge, and medical and health reasoning knowledge [31].

CM intelligent service refers to the utilization of advanced information technologies such as big data, artificial intelligence, and cloud computing to organize, aggregate, analyze, and provide guidance for CM big data, including historical cases, data on well-known doctors and prescriptions, CM literature, health examination data, and internet health data. These services are tailored to specific clinical scenarios and provide accurate personalized and dynamic pharmaceutical services across multiple scenarios, organizations, and devices for the entire lifecycle.

### 2.2. The Connotation of Smart CM Knowledge Services

CM intelligent knowledge is a critical technology for CM services, which are characterized by the homogenization of medical service, the intelligence of knowledge services, the integration of medical education and research, and the precision of service.

#### 2.2.1. The Homogenization of Medical Service

CM services require doctors to accumulate a vast amount of knowledge and experience. However, there are significant differences in the expertise levels between younger and senior doctors, and between doctors from remote and medically advanced areas, resulting in inconsistent CM services. CM intelligent services can effectively leverage the high-quality resources of renowned Chinese medicine hospitals and enhance the capacity for community and grassroots hospitals to accommodate more patients. Integrating medical resources within a region or professional field achieves sharing of high-quality resources and hierarchical diagnosis and treatment. Furthermore, the formation of uniform clinical pathways, quality standards, and evaluation systems regulates the diagnosis and treatment behavior and service quality of hospitals at all levels. To improve clinical efficacy, Xuzhou Affiliated Hospital of Nanjing University of Traditional Chinese Medicine and Xuzhou Hospital of Traditional Chinese Medicine carry out homogeneous dialectical treatment with CM characteristics.

#### 2.2.2. The Intelligence of Knowledge Services

CM knowledge comes from various sources and is highly fragmented, with a lack of logical connections among data. CM intelligent knowledge service requires the rapid correlation and standardization of massive amounts of cross-organizational CM data to organically organize the “knowledge fragments” in the CM field and form a richly expressive and highly extensible CM knowledge system that interconnects concepts and knowledge points. Establishing digital knowledge and case libraries and using technologies such as the internet, cloud computing, and big data enables CM knowledge browsing, retrieval, editing, navigation, and visualization, providing a comprehensive CM knowledge view for CM workers, decision-makers, managers, health professionals, and the public. Based on new-generation information technologies such as machine learning, it provides precise and intelligent knowledge services, such as similar case matching, evidence-based medicine assistance, data analysis, and knowledge recommendation services, to assist in policy-making, medical research, and clinical decision-making. The Institute of Traditional Chinese Medicine Information of the Chinese Academy of Chinese Medical Sciences has built large-scale knowledge systems, such as the CM knowledge maps for health preservation, clinical knowledge, and characteristic therapy, to provide support for knowledge management, knowledge services, education, and training in the field of CM.

#### 2.2.3. The Integration of Medical Education and Research

Through the CM intelligent knowledge service platform, a scientific, educational, and research collaboration network can be established to promote communication and collaboration among research personnel from different institutions, fields, and levels. By combining CM knowledge with modern technological methods, advanced techniques such as data mining, artificial intelligence, and cloud computing can be used to digitize and standardize the study and service of CM theory and practice, continuously improving the scientific and accurate knowledge of CM. The combination of CM scientific research and education can promote the development of modern CM and the inheritance and innovation of CM theory and practice. The China Academy of Chinese Medical Sciences and Shanghai University of Traditional Chinese Medicine jointly carry out personnel training to promote the simultaneous development of clinical practice and scientific research innovation.

#### 2.2.4. The Precision of Service

Different organizations and roles have significant differences in their knowledge needs, which change with task demands, age, health status, and other factors. CM intelligent knowledge services can perceive and model dynamically changing information in real time, achieving accurate knowledge matching and meeting personalized service needs throughout the entire process. The precision of CM intelligent knowledge services is reflected in providing personalized treatment recommendations and medication plans for patients based on their medical conditions, personal traits, and medication history, which improves the diagnostic and treatment abilities and efficiency of doctors, reduces the cost of trial and error for patients, and minimizes treatment risks [32]. Academician Xiaolin Tong proposed state-target dialectics, which combines the traditional dialectical thinking of CM with modern pharmacological research, aiming to improve the precision of CM.

## 3. The Evolution Process of CM Knowledge Services

In this section, we employ bibliometric methods to visualize and summarize the research status of intelligent knowledge services in the field of CM. The bibliometric analysis method extracts tacit knowledge from a large amount of literature data by using data analysis tools, and uses statistical methods to analyze and summarize the fundamental nature and development direction of a certain subject [33]. We use CiteSpace and Excel tools to statistically process the literature data and present the results in the form of tables. The CiteSpace tool is used to analyze relationships such as cooperation and co-occurrence, and draw a visual knowledge map.

In CiteSpace, the overall size of a node indicates the frequency at which the node appears. The node is composed of annual rings of different colors, and each annual ring corresponds to a different time zone and is represented by a different color. The lines between the nodes represent the associations between the nodes. Centrality refers to the intermediary role played by a node on information transfer between other nodes. The higher the centrality of a node, the more important the node is in the process of information transmission [34].

### 3.1. Data Collection

This study used the SCI-E, SSCI, CPCI-S, ESCI, CCR-E, and IC indexes in Web of Science as the data source, and employed advanced search methods to search for #1 and #2, where #1 represents “Chinese medicine” and #2 represents keywords related to intelligent knowledge services. Specifically, #1 is TS = (“traditional Chinese medicine”), and #2 is TS = (“knowledge service” or “intelligent service” or “smart service” or “knowledge discovery” or “knowledge reasoning” or “knowledge recommendation” or “knowledge aggregation” or “knowledge integration” or “knowledge mining” or “knowledge graph” or “knowledge map” or “artificial intelligence” or “knowledge system*” or “knowledge base*”). The time span used for this study was from 2004 to 2023, and the search cutoff date was 10 February 2023. After removing irrelevant parts and duplicates, a total of 1686 relevant articles were retrieved for this time period.

### 3.2. Time Distribution Map of CM Intelligent Knowledge Service

**Figure 1:**
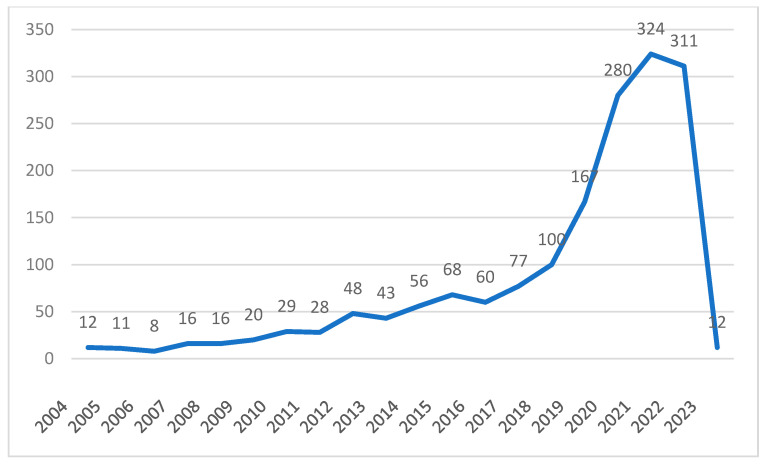
Time-series plot of publication count.

Figure 1 illustrates the change over time in the number of articles published in the field of CM related to intelligent knowledge services. Between 2004 and 2008, research on CM in the field of intelligent knowledge services was at its initial stage of development. There are relatively few theoretical and methodological research results for CM knowledge in digitization and intelligence, and no more than 20 academic papers are published each year. From 2009 to 2017, the number of research results on CM intelligent knowledge services fluctuated slightly, but the overall trend was a gradual increase, with a more significant growth rate. Between 2018 and 2021, research on CM intelligent knowledge services developed rapidly, with a substantial increase, and the research theory and methods were relatively mature, reaching a peak of 324 articles in 2021. The number of articles published in 2022 decreased slightly. The research results for 2023 are from only January and February, with a relatively small number of retrieved papers, so their analysis is not presented in this article.

### 3.3. Space Distribution Analysis

**Figure 2:**
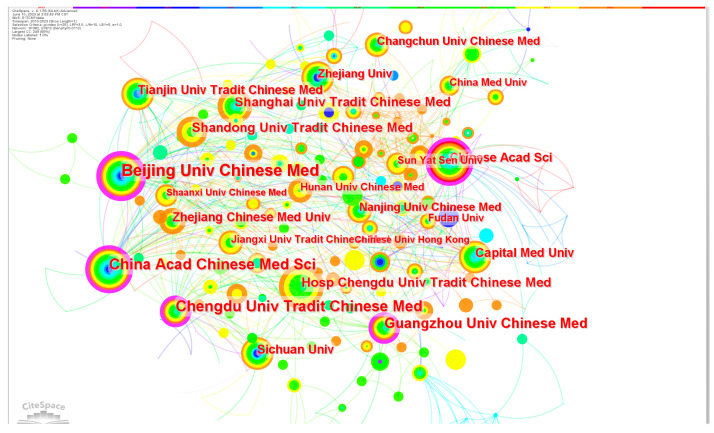
Institutional cooperation network related to CM knowledge service.

**Figure 3:**
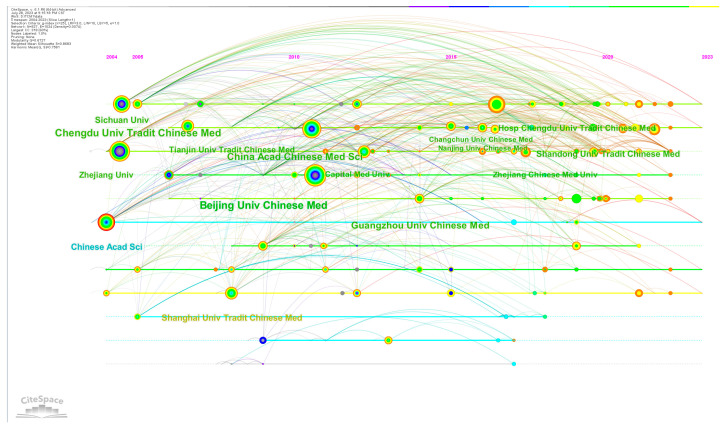
Institutional cooperation timeline chart.

In our study, we analyzed the publication trends of CM knowledge services across 381 institutions. As depicted in Table 1, the average number of articles per institution was 4.42. Notably, the top 20 institutions stood out, collectively publishing 961 articles with an average of 48 articles per institution, surpassing significantly the overall average. Beijing University of Chinese Medicine, Chengdu University of Traditional Chinese Medicine, and China Academy of Chinese Medical Sciences emerged as the three most prolific institutions in terms of publication volume, indicating their prominent position within the field. These institutions also exhibited high centrality. Furthermore, assessing the level of collaboration between research institutions serves as an important indicator for evaluating the research landscape in a specific domain [35]. As shown in Figure 2, it demonstrates that there is close cooperation among the institutions. Figure 3 shows the timeline for institutions to start research on CM knowledge services.

**Figure 4:**
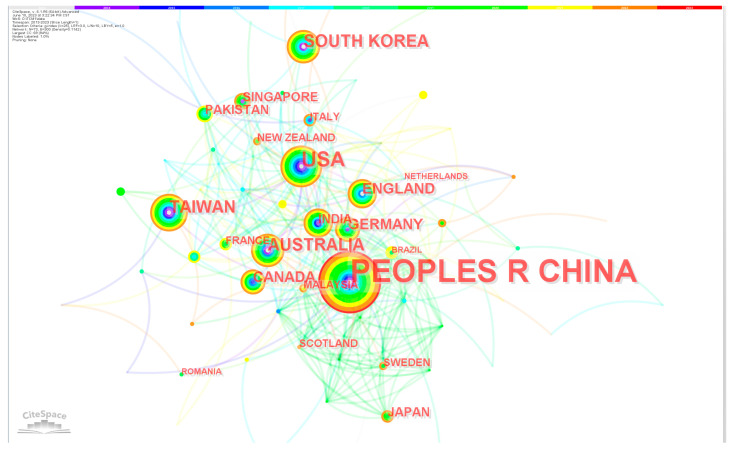
Country and region cooperation network related to CM knowledge service.

**Figure 5:**
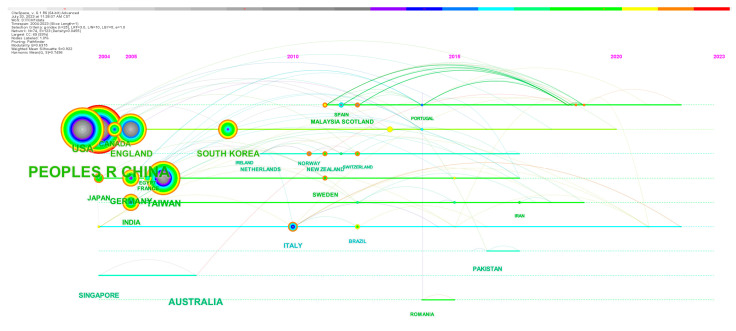
Country and region cooperation timeline chart.

Next, we analyzed the countries/regions that published relevant articles in this field and generated a network of country collaborations, as shown in Figure 4. Therefore, we can find that countries/regions cooperate closely, especially countries with a large number of publications. Figure 5 shows the timeline of countries/regions starting research on CM knowledge services. As shown in Table 2, among the top 20 countries or regions (as shown in the table) of knowledge service research on CM, 8 are in Asia, including China, Taiwan (China), South Korea, India, Singapore, Pakistan, Japan, and Malaysia; 7 in Europe, including the United Kingdom, Germany, Italy, France, Sweden, Scotland, and Romania; in addition, there are the United States and Canada in North America, and Brazil in South America. There is no doubt that China is the country with the largest number of studies on CM, far exceeding other countries and regions, accounting for 69.1% of all publications. Centrality indicates the importance of a node, and among the 20 countries with the largest number of publications, China has the highest centrality, followed by the United States.

### 3.4. Evolutionary Analysis of Hot Topics

**Figure 6:**
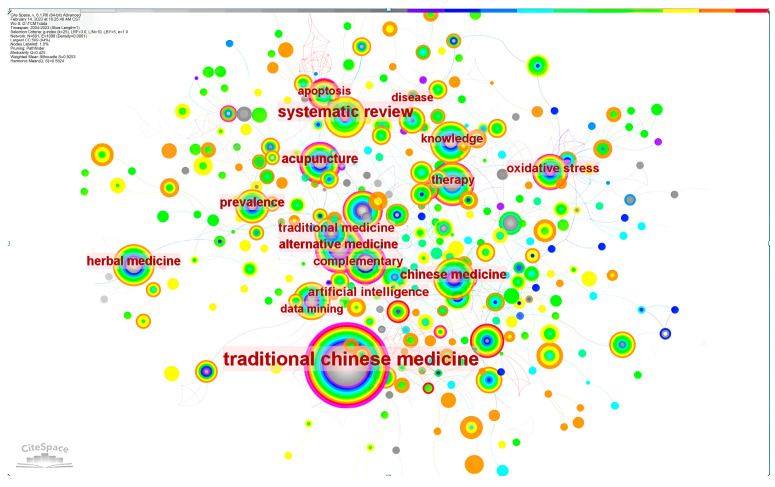
Co-occurrence network of keywords.

**Figure 7:**
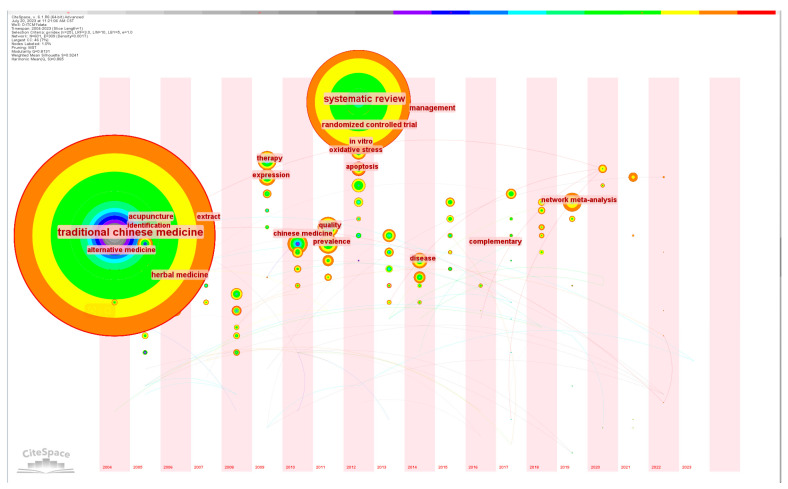
Co-occurrence time chart.

Keywords are highly concise and general about an article. By analyzing high-frequency keywords, we can understand popular research topics in this field. Important keywords, as shown in Table 3, include “traditional Chinese medicine”, “systematic review”, “acupuncture”, “prevalence”, “alternative medicine”, “complementary”, and “artificial intelligence”. It can be seen from Figure 6 that there is a strong connection between keywords, which indicates that most of the research in the field of CM knowledge services is multisubject. Figure 7 shows the timing of keyword co-occurrence. Keywords related to knowledge services include systematic review, knowledge, data mining, etc., indicating that most of the current research on knowledge services in CM is a systematic summary of previous knowledge and knowledge mining. In view of the combination of new technologies and products such as cloud computing and artificial intelligence derived from internet big data and CM, the modernization of CM is accelerating to achieve leapfrog development, which puts forward higher requirements for CM knowledge services. The research results show that the new generation of information technology can be combined with the academic thinking of CM [36] to provide knowledge services in various aspects such as pharmacological analysis [37], auxiliary diagnosis and treatment [38], and optimization of the diagnosis process [39].

**Figure 8:**
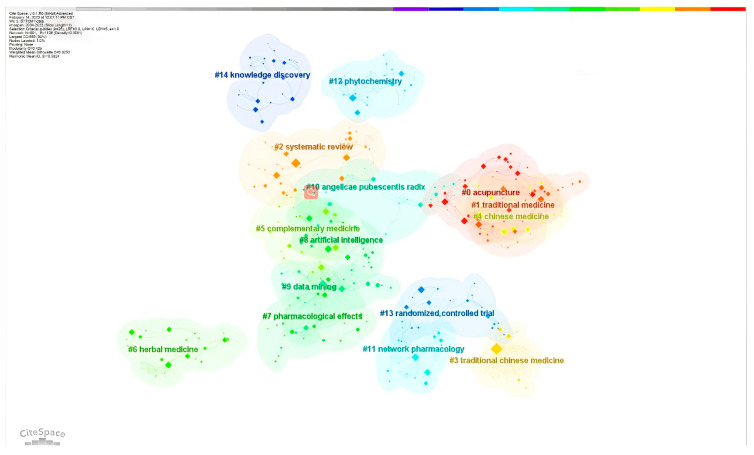
Cluster analysis of keywords.

**Figure 9:**
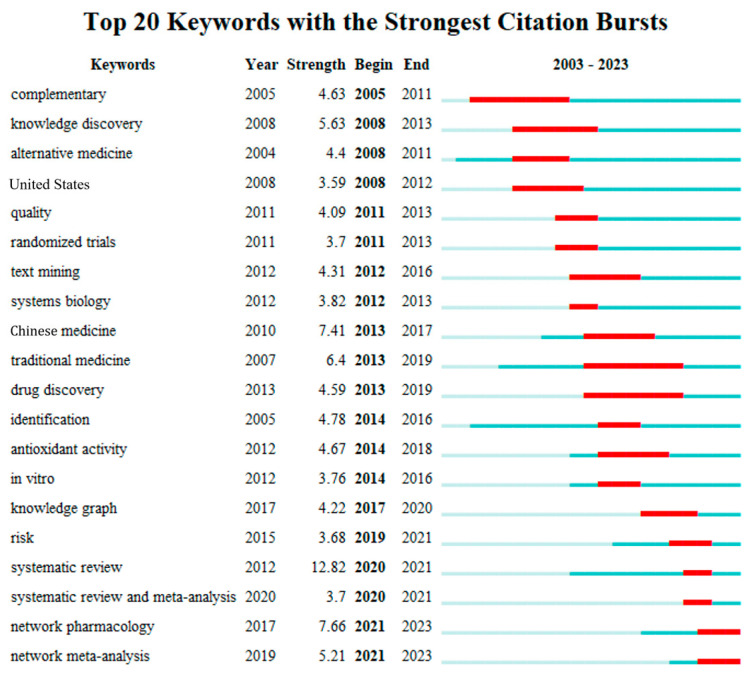
Keywords with the strongest citation bursts.

By studying the co-occurrence cluster analysis of these keywords, as shown in Figure 8, the research hotspots are mainly focused on the mechanisms of CM, knowledge mining and discovery in CM, application of artificial intelligence in CM, and alternative therapies. As shown in Figure 9, the thematic changes in research on intelligent CM knowledge services over the past 20 years can be observed through keyword clustering and emergent keywords. Strength refers to the burst strength of keywords. It can be seen from the words “complementary” and “alternative medicine” that CM often serves patients as a supplement to modern medicine. Yang et al. pointed out that CM is a good alternative to modern medicine because of its many targets and few side effects [40]. The prominence of words such as “randomized trials”, “systems biology”, “identification”, “antagonistic activity”, and “network pharmacology” over the past 20 years indicates the interest of scholars from various countries concerning CM mechanisms and the standardization and internationalization of CM data [28,29,30]. However, the diagnosis and treatment process of CM is based on the theoretical system of Chinese medicine by examining the condition, determining the type of disease, distinguishing symptoms, and using the method and viewpoint of dialectical treatment to treat the disease. Research on pharmacology or pathology alone is insufficient to cover the knowledge content of CM. Terms such as “knowledge graph”, “data mining”, “systematic review”, and “meta-analysis” indicate that organizing CM knowledge is usually done from a holistic perspective, such as association with CM philosophy, CM physiology, etiology, and pathogenesis. With the vigorous development of the internet, the application of new-generation information technologies such as big data and artificial intelligence in CM diagnosis has made CM diagnosis more quantitative, objective, and standardized [41], and the development of precise CM knowledge services is an inevitable requirement for the modernization of Chinese medicine. At the same time, it is necessary to maintain the dialectical characteristics of CM.

## 4. The Smart CM Services under the 5P Healthcare Mode

**Figure 10:**
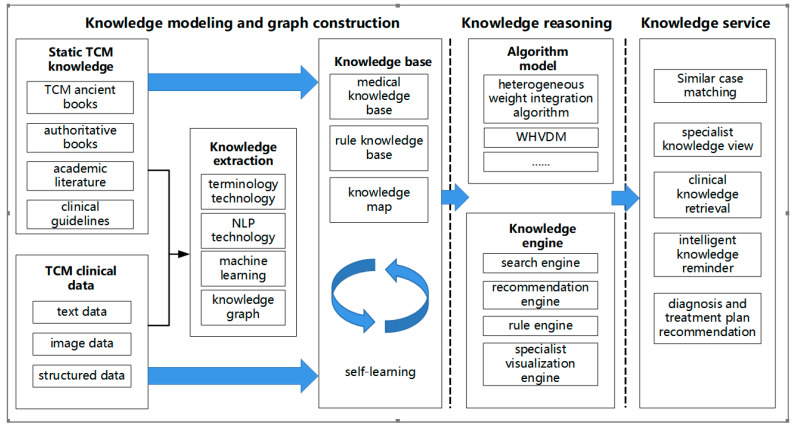
Framework of smart CM knowledge services.

The new model of CM service, driven by big data, can address the challenges of CM development under new circumstances. The 5P medical mode mentioned above refers to preventive, predictive, personalized, participatory, and precision medicine. Specifically, preventive refers to the early prevention of disease risks that have not occurred, and predictive refers to predicting the occurrence and development of diseases and uncovering changes in health status. Personalized refers to individualized medicine, including individualized diagnosis and individualized treatment. Participatory means that each individual should be responsible for their personal health and actively participate in disease prevention and health promotion. Precision medicine refers to the practice of personalized multidisciplinary comprehensive treatment. The practice of precision medicine should be a patient-centered, open, medical cognition and practice process that keeps pace with the times and is constantly improving. Compared with general medical knowledge services, there are certain differences in knowledge sources, knowledge systems, and theoretical thinking modes among traditional Chinese medicine knowledge services [42], as shown in Table 4. Under the 5P medical mode, the CM smart service model places the patient at the center of a new medical model that combines CM theory with the 5P medical mode. The CM knowledge smart service model can track changes in the patient’s body in real time and adjust the CM implementation plan promptly. By providing a full-cycle smart pharmacy service, including the organization and dynamic updating of medical case knowledge, knowledge generation and discovery based on case reasoning, as well as knowledge service recommendations considering comprehensive utility and diversity, the CM knowledge smart service model provides decision support for CM doctors and helps patients obtain the best treatment plan. This study proposes a basic framework for the CM knowledge smart service model driven by data and knowledge under the 5P medical mode, as illustrated in Figure 10.

The framework of the CM knowledge intelligent service model includes three parts: knowledge organization, knowledge generation, and knowledge service. Knowledge organization mainly includes case base construction, knowledge modeling, and picture construction. Knowledge generation mainly includes reasoning knowledge, and knowledge service mainly includes case knowledge recommendation, professional knowledge display, and other related services. This model is aimed at improving medical and health decision-making, hospital management, clinical teaching, and scientific research. To achieve this, a medical knowledge base and graph are built using authoritative books, academic literature, clinical pathways, and diagnosis and treatment guidelines. The knowledge base is constantly updated through a self-learning mechanism. Additionally, an example library is created using medical and health big data, and different algorithm models are applied for knowledge generation and discovery in different management decision-making scenarios. Through matching, retrieval, recommendation, reminder, view navigation, and other methods, the framework provides knowledge services to users.

### 4.1. The Organization of CM Knowledge

Case knowledge is the foundation of CM knowledge, because it contains a vast amount of expert knowledge that is difficult to quantify scientifically but is crucial in providing decision-making information support for the CM medical process. CM doctors use the “observation, listening, questioning, and pulse diagnosis” method to collect clinical data from patients. This approach relies on four aspects of data collection: vision, smell, auscultation, and palpation. By comprehensively analyzing these four aspects of data, CM doctors differentiate syndromes and determine the etiology, location, nature, and pathogenesis of diseases. AI-assisted CM diagnosis relies heavily on data from these four diagnostic methods [43]. For instance, observation can collect data using modern medical imaging techniques (such as CT, MRI, and so on) or in the form of pictures, recording the patient’s skin color, facial features, tongue coating, and tongue quality, among others. Listening records patient data information in text, such as the patient’s bad breath, body odor, sweat odor, and so on. Questioning records the inquiry information in text or audio. Pulse diagnosis records data through wearable devices or other sensors, or by manually taking the patient’s pulse and recording text data. Given the vast amount of heterogeneous data from multiple sources in CM case knowledge, it is essential to effectively organize and manage medical and health case knowledge to achieve fine-grained management and accurate services. Moreover, CM data involves patients’ private personal information and medical institutions’ business secrets, prompting the need to ensure the security and confidentiality of data. Cross-domain CM data security sharing is an urgent problem that needs to be addressed.

#### 4.1.1. CM Case Knowledge Organization Based on Key Clinical Features Extraction

**Figure 11:**
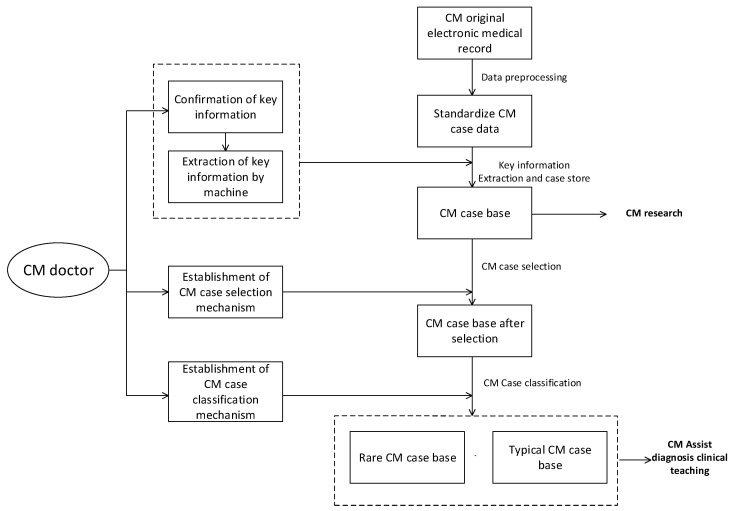
Flowchart for building the case base.

In the context of CM medical and health management decision-making, CM experts rely on clinical pathways, diagnosis guidelines, and disease consensus to determine the main characteristic attributes, conclusions, and solution categories of cases. They then use a CM case automatic generation algorithm, which integrates natural language processing and key information extraction, to organize CM case knowledge. To evaluate the quality of CM cases, the system establishes a two-stage dual evaluation mechanism of “storage-use” + “quality-usability.” Only cases with good evaluations from doctors can enter the case bases, and high-quality cases can be further classified to build rare-disease case bases and well-known-doctor case bases to meet the knowledge service needs for different CM scenarios. The flowchart for building the CM case base is shown in Figure 11.

To extract key feature information, natural language processing methods are used based on authoritative disease knowledge provided by doctors. A pretrained medical field word vector dictionary is then used to obtain a word vector matrix of unstructured text data. This matrix is then input into multiple prebuilt segmenters to obtain segmented sentence sequences, which are in turn input into multiple prebuilt part-of-speech taggers to obtain part-of-speech tagging results. Using these results, the key information is obtained, which is then fused and matched based on expert experience to obtain the key case information and form case knowledge.

The first loss function during the pretraining process of the built segmenter is

(1) $$Loss1=\frac{1}{P}\sum_{i=1}^{P}\left(1-h_{true}^{\left(c^{\left(p\right)}\right)}\right)$$

where $h_{true}^{\left(c^{\left(p\right)}\right)}$ represents the probability value corresponding to the correct character label, $h_{true}^{\left(c^{\left(p\right)}\right)}\in [0,1]$; *P* represents the total number of characters; and *p* represents the *p*th character.

The second loss function during the training process of the prebuilt part-of-speech tagger is

(2) $$Loss2=\frac{1}{Q}\sum_{i=1}^{Q}\left(1-e_{true}^{\left(w^{\left(q\right)}\right)}\right)$$

where $e_{true}^{\left(w^{\left(q\right)}\right)}$ is the probability value of the correct part-of-speech tag, $e_{true}^{\left(w^{\left(q\right)}\right)}\in [0,1]$; *Q* represents the number of words after sentence segmentation; and *q* represents the *q*th word after segmentation.

The calculation of the overall loss function is as follows:

(3) Loss=Loss1+Loss2

The CM knowledge organization system is capable of summarizing and generalizing high-quality case evaluation criteria from historical evaluation data. Through reinforcement learning methods, it automatically learns and forms high-quality case evaluation rules while controlling the case size and continuously injecting high-quality cases and gradually eliminating low-quality cases. This continuous improvement in case quality avoids unlimited expansion and ensures dynamic updates of case knowledge, thereby providing the possibility for large-scale case quality evaluation.

#### 4.1.2. Medical Data Security Sharing Based on Horizontal and Vertical Federated Learning

Cases contain a significant amount of sensitive information, and in recent years, data privacy protection has become an increasingly important concern for society. The exchange of data between enterprises and organizations without user authorization is strictly prohibited. Data sharing between different CM hospitals and clinics is difficult, resulting in various “data islands” of different sizes, which poses significant challenges to AI and machine learning. In the medical and health field, accurate results can be obtained only after analyzing a large amount of data and cases. However, because of the unique nature of medical big data and the differences in information collection systems used by various hospitals, different types of medical data cannot be easily exchanged. Data sharing between hospitals is challenging, and sharing data between other health and elder-care institutions is even more difficult. By sharing user data between different institutions without compromising privacy, more comprehensive analysis data can be obtained, which can assist decision-makers in making informed judgments.

Federated learning is a distributed machine learning algorithm that facilitates secure management of cross-organizational and heterogeneous medical and health data resources. The algorithm involves several key steps. First, based on the first and second data sharing requests initiated by the first institution on the health data resource sharing platform, the second and third institutions that respond to these requests are determined. Next, the relevant parameters of the first model built by the first institution on the data sharing platform are updated using horizontal federated learning algorithms, based on the relevant health data resources of the second institution. The third step involves updating the parameters of the second model built by the first institution on the data resource sharing platform using vertical federated learning algorithms, based on the health data resources of the third institution. In the fourth step, the Shapley value is used to allocate model construction, which helps determine the training results from the first and second models obtained by the first institution. Finally, based on the training results of the first and second models, and in conjunction with the relevant health data resources of the first institution, secure aggregation of medical and health big data across organizations is achieved. The incentive parameters for each participating institution in the allocation modeling process can be expressed by Formula (4).

(4) $$\varphi_{i}\left(v\right)=\sum_{S\subseteq N\backslash \left\{i\right\}}\frac{S!\left(N-S-1\right)!}{N!}\left(v_{\left(S\cup \left\{i\right\}\right)}-v_{S}\right)$$

where $\varphi_{i}\left(v\right)$ represents the incentive parameter of the *i*-th participating institution; *N* represents the total number of participating institutions; *S* represents a subset of the *N* participating institutions; $v_{S}$ represents the individual contribution value of the subset *S*; $v_{\left(S\cup \left\{i\right\}\right)}$ represents the contribution value of the set *S*; and $N\backslash \left\{i\right\}$ represents the subset that does not include the *i*-th participating institution.

### 4.2. CBR Method for Health Knowledge Generation and Discovery

CBR is an important subfield of artificial intelligence that serves as an effective means for organizing knowledge. CBR uses big data to organize cases and solves new management decision problems by matching them with the most similar historical cases and using expert knowledge gathered from those past cases. The reasoning process of CBR closely resembles that of human decision-making [29]. When doctors encounter new problems, they often recall past experiences managing similar situations and adjust and modify that information to formulate solutions to the current problem. Furthermore, case-based reasoning is a flexible knowledge reasoning technique that can flexibly construct case libraries and provide knowledge services based on different management decision tasks and real-time temporal information, making it well suited for organizing CM case knowledge. CM case knowledge is often ambiguous and difficult to verify in the diagnosis process. Case-based reasoning can efficiently solve problems without starting from the beginning by reusing historical knowledge, significantly improving problem-solving efficiency and providing more complete and interpretable initial solutions.

Case knowledge is the core of CM knowledge and can provide useful decision-making information support for the medical process of CM. Reasoning, generation, and discovery based on medical big data, CM case knowledge, and general medical knowledge can provide fine-grained management methods for hospital-assisted decision-making and risk warning. This section mainly introduces a human–machine collaborative case knowledge generation method and a case knowledge discovery method that considers implicit feedback.

#### 4.2.1. Human–Computer Collaborative Method for CM Case Knowledge Generation

A human–machine collaborative medical and health knowledge generation method is proposed, which is aimed at case knowledge bases and general knowledge bases, to achieve the knowledge generation method of new medical decision-making solutions. By matching, reusing, modifying, quality evaluation, and review of historical cases, the knowledge generation of new medical decision-making solutions is achieved, and human participation is used to evaluate case quality and improve case knowledge, effectively reducing medical risks [44].

Cases are represented by (*x*, *y*) vectors, where $x=(x_{1},x_{2},\ldots ,x_{n})$ is a vector of independent variables that represent the feature attributes, and *y* ∈ *Y*, where *Y* is a discrete variable corresponding to the class. In the case library of solved historical cases, the class values are known. Therefore, when given a new unsolved target case, it can be retrieved by finding the most similar case in the library. To generate a solution for the new case, the weighted heterogeneous value distance measure (WHVDM) is used for case matching, providing a knowledge reference for decision makers.

Specifically, the WHVDM between the new target case t and the stored case r is defined as

$$WHVDM\left(t,r\right)=\sqrt{\sum_{i=1}^{n}w_{i}d_{i}^{2}\left(t,r\right)}$$

where

(5) $$d_{i}^{2}\left(t,r\right)=\left\{\begin{array}{c} vdm\left(t,r\right),\mathrm{if}\;x_{i}\;\mathrm{is}\;\mathrm{discrete}\\ diff^{2}\left(x_{t,i},x_{r,i}\right),\mathrm{if}\;x_{i}\;\mathrm{is}\;\mathrm{continuous} \end{array}\right.$$

In Equation (5), *vdm*(*t*,*r*) is the value difference measure (VDM) proposed by Stanfill and Waltz. The VDM between the discrete attribute *x_i_* of the target case *t* and the stored case *r* is defined as

(6) $$vdm_{i}\left(t,r\right)=\sum_{a\in Y}\left(\mathrm{Pr}(y=a|x_{i}=x_{t,i}\right)-\mathrm{Pr}(y=a\left|x_{i}=x_{r,i}\right){)}^{2}\;•\sqrt{\sum_{a\in Y}\mathrm{Pr}(y=a|x_{i}=x_{t,i}{)}^{2}}$$

In Equation (5), ${diff}^{2}\left(x_{t,i},x_{r,i}\right)$ is the Euclidean distance measurement, which is part of various distance measurements used in CBR systems. Specifically, given a new target case *t* and a stored case *r*:

(7) $${diff}^{2}\left(x_{t,i},x_{r,i}\right)={{(x}_{t,i}-x_{r,i})}^{2}$$

This method is suitable for measuring the distance between cases that contain both discrete and continuous variables, highlighting the impact of the relative importance of case attributes. This impact is reflected in the weights of the feature attribute vector, which are obtained through a genetic algorithm [45]. Compared with commonly used knowledge discovery methods such as PBF neural network, CART, logistic regression, and naïve Bayes, this method has an accuracy improvement of over 3.2% and at least a 4.5% improvement in the comprehensive F-value performance evaluation index [46].

#### 4.2.2. A Case Knowledge Discovery Method Considering Implicit Feedback in Human–Computer Interaction

As big data technology and medical digital systems continue to mature, the interaction behavior information between doctors and systems, such as personal preferences and case evaluation scores, is recorded in databases, providing huge data support for data mining. User feedback information is mainly divided into explicit feedback data and implicit feedback data. Explicit feedback behavior is reflected mainly through case rating, collection, labeling, and other methods, whereas implicit feedback behavior refers to the personal preferences of doctors, and obtaining and using such data may involve a certain delay. Considering doctors’ implicit behavior in case knowledge discovery can predict the most needed case knowledge based on the browsing preferences and behavior sequences of CM doctors, which is of great significance for personalized configuration of doctor users, improving work efficiency, as well as diagnosis and treatment levels.

The main process is as follows: The browsing sequence and rating records of the attending physician are preprocessed, and the similarity between each case is calculated. Then, the generalized matrix factorization (GMF) and multilayer perceptron (MLP) neural networks are used to extract the physician’s behavior characteristics and habits from the sequence, and combined with the rating information of past cases to obtain preliminary recommendation results. Finally, after personal screening by the doctor, the final recommendation list is obtained.

Specifically, the user–case interaction matrix Y obtained from implicit feedback is defined as

(8) yui=10

where a value of 1 means that there is human–computer interaction between user and *i*; a value of 0 may mean that user does not like *i*, or that user does not know that there is *i* at all.

After dimensionality reduction in the embedding layer, the input binary sparse vector is mapped to a dense vector, and then with the help of the GMF model, the inner product of the user latent vector and the case latent vector is used as the evaluation of the user’s preference for *i*, and the first neural collaborative filtering (NCF) layer is defined:

(9) $$\phi_{1}\left(p_{u},q_{i}\right)=p_{u}\circ q_{i}$$

where $p_{u}=P^{T}v_{u}^{U},q_{i}=Q^{T}\nu_{i}^{I}$ are latent vectors of users and cases, respectively, projected onto the output layer:

(10) $${\hat{y}}_{ui}=a_{out}\left(h^{T}\left(p_{u}\circ q_{i}\right)\right)$$

where $a_{out}$ is the activation function of the output layer, selected by the ReLU function, and *h* is the weight of the output layer, which is obtained through training.

Next, with the help of the MLP model, we use the standard multilayer perceptron to learn the potential features of users and cases, and calculate the output for each layer of MLP under the NCF framework:

(11) $$z_{i}=\{\frac{a_{i}\left(W_{L}^{T}z_{lL-1}+b_{L}\right),i=2\cdots L}{\varphi_{i}(p_{u},q_{i})=[\frac{p_{u}}{q_{i}}],i=1}$$

The output of the final model is

(12) $$y_{ui}=\sigma \left(h^{T}z_{L}\right)$$

The knowledge recommendation results are obtained by splicing the last layer of the hidden layers, applying the exponential mechanism for privacy preservation, and then normalizing the resulting probability values.

### 4.3. Dynamic Personalized Knowledge Recommendation

The CM knowledge smart service platform integrates data resources from various fields and caters to different levels of CM health service demands, including the government, medical consortiums, hospitals, and individuals. By leveraging the behavior characteristics and patterns of different patient groups, it aggregates real-time sensing information and historical health data to track health status and demands. The platform provides personalized, dynamic, visualized, and intelligent panoramic health knowledge services for different groups and scenarios under the 5P medical mode. It achieves this through the use of an individual health assessment model and demand evolution model based on real-time time-series health data.

#### 4.3.1. Health Risk Assessment Based on Time-Series Warning Signals

Through the analysis and processing of relevant medical information data, the changing characteristics and patterns of the health status of different patients can be studied, allowing for accurate identification of disease clues and health risks. With this information, personalized health assessment and demand evolution models can be established based on real-time time-series health data. This enables the provision of a service that recommends intervention plans based on users’ characteristics, ultimately meeting the individualized health needs of patients.

The specific process is as follows: When an early warning signal is given, user tag vectors are constructed based on the user’s physical condition, and collaborative filtering based on singular value decomposition is performed. After obtaining a plan, the BP-DS model is used to match the plan details with the user’s physical characteristic values and adjusts them accordingly. Finally, trend fitting is applied to predict the user’s physical condition and decide whether to end the treatment or choose the next stage of the plan, thereby generating a complete intervention plan.

Specifically, the process involves using the Pearson correlation similarity calculation to obtain a matrix that represents the similarity between users:

(13) $${Similarity}_{r}\left(u,v\right)=\frac{\sum_{i\in C}\left(r_{u,i}-\bar{r_{u}}\right)\left(r_{v,i}-\bar{r_{v}}\right)}{\sqrt{\sum_{i\in C}(r_{u,i}-\bar{r_{u}}{)}^{2}}\sqrt{\sum_{i\in C}(r_{v,i}-\bar{r_{v}}{)}^{2}}}$$

Calculate the preference of user tag attributes and obtain the similarity of attribute preferences:

(14) $$P_{ui}=\frac{Weight_{ui}}{Weight_{u}}$$

(15) $${Similarity}_{s}\left(u,v\right)=\frac{\sum_{i=1}^{k}p_{ui}p_{vi}}{\sqrt{\sum_{i=1}^{k}p_{ui}^{2}}\sqrt{\sum_{i=1}^{k}p_{vi}^{2}}}$$

The comprehensive similarity is determined by taking into account both the similarity between users and the similarity of attribute preferences:

(16) $$Similarity\left(u,v\right)=w\times {Similarity}_{r}\left(u,v\right)+\left(1-w\right)\times {Similarity}_{s}\left(u,v\right)$$

The user rating matrix and the project tag attribute matrix T are used in calculations to obtain the predicted value for the user:

(17) $${\mathrm{Pre}}_{u,i}={\bar{R}}_{u}+\frac{\sum_{v\in K-\mathrm{neighbours}}Similarity\left(u,v\right)\times \left(R_{v,i}-{\bar{R}}_{v}\right)}{\sum_{v\in K-\mathrm{neighbours}}Similarity\left(u,v\right)}$$

#### 4.3.2. A Collaborative Recommendation for Medical Research and Education Integration

The medical education and research integration model refers to the application of the industry–university–research cooperation model in higher medical education. The CM intelligent knowledge service meets the needs of doctors and hospitals for medical treatment, education, and research integration. On the one hand, it provides doctors with decision-making assistance solutions by recommending similar cases. On the other hand, it provides classic clinical teaching cases and relevant common knowledge to young and grassroots doctors who lack experience in diagnosis, treatment, research, and training, thereby offering knowledge services.

**Figure 12:**
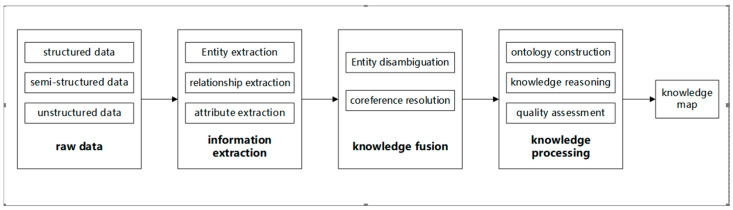
Flowchart for constructing the knowledge graph.

This method uses a vast amount of clinical cases and general CM medical knowledge, and applies them to the medical education and research model to proactively recommend relevant medical and health knowledge based on dynamic perception of patient health status and doctor needs. The specific process involves first extracting entities and corresponding concepts using a hierarchical segmentation processing algorithm, and constructing a mapping relationship between them to obtain the medical knowledge base in the form of an <entity, concept> binary tuple. When a new patient record appears, features are extracted from the case information using the term frequency and inverse document frequency (TF-IDF) method to build the case feature set, which is then matched with the preset clinical case library to obtain similar cases. The LSTM-CRF named entity recognition technology is then used to extract medical terms, which are associated with the general CM medical knowledge base to provide detailed medical knowledge pages. The medical knowledge recommendation content is displayed with the help of the medical knowledge graph, to achieve the purpose of knowledge learning or disease research. Figure 12 shows the flowchart for constructing the knowledge graph.

## 5. Innovative CM Knowledge Services Models in the Era of Digitalization

At present, the theory and practice of intelligent CM knowledge services are in their early stages. To address this, an interdisciplinary approach can be used to develop and utilize a CM knowledge service system driven by big data under the 5P medical mode. This system will be unique to China and internationally leading, and it will aid in advancing the scientific research level of CM health management in China to the forefront of the world. In addition, it will provide much-needed scientific theoretical support for CM knowledge services.

### 5.1. Case-Based CM Knowledge Service Model Guided by Holistic View and Dialectical

A significant part of CM knowledge is implicit, originating from the clinical practice of CM practitioners and unique to each individual, making it challenging to describe and teach using language and text. This is known as “implicit knowledge”. Because of the existence of implicit CM knowledge, the transmission of CM is often achieved through observation, imitation, communication, and practical experience. The process of exploring, transmitting, and sharing implicit CM knowledge involves transforming implicit knowledge into explicit knowledge and integrating various resources, such as well-known doctors, disciplines, and information [47].

In managing implicit CM knowledge, a holistic view and dialectical approach should be used as guidance, and the diagnostic and reasoning processes of well-known doctors should be fully preserved. Effective clinical information should be obtained from a vast amount of CM knowledge to avoid knowledge deviation, omission, and distortion in the transmission of CM. This is essential for the preservation, sorting, and sharing of implicit CM knowledge. Therefore, it is urgent to construct a CM knowledge base centered on well-known CM cases, fully preserving the diagnostic and reasoning processes of well-known doctors and updating the case library automatically through self-learning and adaptive mechanisms.

### 5.2. Human–Machine Cooperative Medical Knowledge Recommendation Service Model

The traditional approach to knowledge reasoning relies mainly on computer models and algorithms, with limited human involvement, making it difficult to comprehend the reasoning results and limiting their practical utility [48]. Compared with the traditional clinical decision support system, the assisted diagnosis and treatment system participated by doctors has the advantages of knowledge adoption, ease of use, usefulness in improving medical quality, satisfaction of system use, interpretability of recommended schemes, perceived security, and ability enhancement have advantages [48]. To improve the acceptability of knowledge reasoning results, it is necessary to overcome the bottleneck of depending solely on machine reasoning for knowledge acquisition. Therefore, there is an urgent need to clarify the production and circulation rules for multisource and multimodal medical and health data, such as diagnosis and treatment data and health data, in the context of CM health services. Establishing a multimodal intelligent knowledge reasoning system can create a human-in-the-loop mechanism for knowledge reasoning and acquisition, providing a new paradigm for CM medical and health knowledge reasoning.

### 5.3. Active Knowledge Service Model for 5P Healthcare

In the era of intelligent interconnection, active health is a medical and health service model that is guided by the concept of holistic medicine and the CM principle of preventing diseases before they occur. It is supported by the new generation of information technologies such as the internet and artificial intelligence, and aims to provide proactive prevention and diagnosis services. The construction and application of a CM knowledge-based intelligent service system emphasizes personalization, precision, participation, and collaboration, with the aim of transforming disease treatment into health management. Therefore, there is an urgent need to conduct research on actively responsive and flexible disease risk judgment mechanisms, personalized treatment plan generation based on comprehensive patient health information, and health information knowledge recommendation mechanisms. Additionally, it is crucial to establish information security sharing and collaboration mechanisms among various medical institutions to provide doctors with high-quality knowledge services, and to improve medical quality and efficiency.

### 5.4. Panoramic and Dynamic Knowledge Service Mode Driven by Knowledge and Data

The 5P medical mode emphasizes the importance of personalized and precise medical services. To achieve dynamic health services that cater to individual needs, it is essential to overcome the limitations in relying mainly on static, local, and limited historical medical and health information for knowledge acquisition. This requires expanding the theoretical system of medical and health services and management, and establishing a new model of panoramic, dynamic, active, and personalized services based on real-time health perception and demand assessment [49]. Therefore, there is an urgent need to construct a dynamic and real-time CM case knowledge base based on CM knowledge and health and diagnostic data. This involves developing intelligent models for health risk assessment and dynamic intervention program recommendations based on time-series warning signals. By breaking down the business barriers and geographic boundaries of CM practitioners, it is possible to transform CM knowledge services from passive treatment and static services to real-time perception, dynamic evaluation, and active intervention, achieving precise and personalized health service modes.

## 6. Conclusions

The CM intelligent knowledge service model, driven by data and knowledge, has significantly transformed the CM knowledge service model, thereby promoting the development of the CM industry. CM is a unique medical system that requires a professional medical background and CM knowledge reserves. CM intelligent knowledge services need to provide accurate and credible content while also respecting the development process and characteristics of traditional medicine. They cannot simply be compared with modern medicine. This article reviews, summarizes, and analyzes the research status of CM intelligent knowledge service models, revealing that new information technologies such as artificial intelligence have been widely applied to CM services. However, the research on the CM knowledge service system is still incomplete. Therefore, this article proposes a CM intelligent knowledge service model under the 5P medical mode, which systematically studies the knowledge organization and data sharing mechanism, knowledge generation and discovery, knowledge reasoning, and full-cycle active personalized service mode of CM intelligent knowledge services. CM knowledge organization, knowledge generation, and knowledge service interact and depend on each other, forming a relatively complete CM knowledge management ecosystem. Through the effective integration and coordination of these three aspects, the innovation, dissemination, and utilization of CM knowledge can be realized, and the competitiveness and innovation ability of CM organizations can be improved. The CM intelligent knowledge service combines CM general knowledge, case knowledge, and reasoning knowledge to serve CM diagnosis, treatment, teaching, and research, which is of great significance for the development of CM and public welfare.

In future research, we should fully leverage the potential of the new generation of information technology to enhance and ensure the quality of CM knowledge service, focusing on the following three key aspects:

1. Facilitating the Integration of CM and Western Medicine:

Both Chinese and Western medicine boast their unique strengths and inherent weaknesses. By amalgamating the concepts and techniques of Chinese and Western medicine, we can foster cross-learning and capitalize on each system’s strengths. This harmonious integration has the potential to yield more comprehensive and effective healthcare solutions [50,51].

2. Unearthing the Untapped Potential of Folk Chinese Medicine:

Folk CM holds a treasure trove of invaluable knowledge, encompassing secret recipes and unique techniques that have been passed down through generations. It is of paramount importance to actively preserve and retrieve this traditional wisdom. Thoroughly researching and documenting folk remedies and practices allows us to incorporate these hidden gems into mainstream CM knowledge, thus enriching the array of healthcare practices and treatments.

3. Enhancing CM Diagnosis and Medication through Standardized and Precise Prediction:

To bolster the accuracy of CM diagnosis and medication, adopting standardized and precise prediction methods plays a pivotal role. Harnessing cutting-edge technologies and data analysis tools enables the optimization of diagnostic processes [52], leading to more accurate treatment plans and improved patient outcomes.

## Figures and Tables

**Table 1:** List of the top 20 institutions with the number of published articles.

	Year	Number of Published Articles	Institution	Centrality
1	2013	134	Beijing Univ Chinese Med	0.21
2	2013	114	Chengdu Univ Tradit Chinese Med	0.11
3	2013	100	China Acad Chinese Med Sci	0.14
4	2014	79	Guangzhou Univ Chinese Med	0.11
5	2020	59	Shandong Univ Tradit Chinese Med	0.05
6	2013	51	Shanghai Univ Tradit Chinese Med	0.05
7	2019	48	Hosp Chengdu Univ Tradit Chinese Med	0.03
8	2013	46	Tianjin Univ Tradit Chinese Med	0.07
9	2013	46	Chinese Acad Sci	0.12
10	2018	39	Zhejiang Chinese Med Univ	0.04
11	2013	38	Sichuan Univ	0.04
12	2014	34	Capital Med Univ	0.08
13	2016	27	Nanjing Univ Chinese Med	0.02
14	2013	26	Zhejiang Univ	0.04
15	2016	24	Changchun Univ Chinese Med	0.01
16	2020	21	Jiangxi Univ Tradit Chinese Med	0
17	2020	20	Hunan Univ Chinese Med	0.02
18	2019	19	Sun Yat Sen Univ	0.02
19	2013	18	Fudan Univ	0.02
20	2013	18	Kyung Hee Univ	0

**Table 2:** List of the top 20 countries/regions with number of published articles.

	Year	Number of Published Articles	Institution	Centrality
1	2013	1270	PEOPLES R CHINA	0.69
2	2013	96	USA	0.26
3	2013	48	AUSTRALIA	0.03
4	2013	42	TAIWAN	0.03
5	2013	37	SOUTH KOREA	0.07
6	2013	28	ENGLAND	0.02
7	2013	25	CANADA	0.01
8	2014	24	GERMANY	0.12
9	2014	20	INDIA	0.19
10	2014	15	SINGAPORE	0
11	2016	14	PAKISTAN	0.09
12	2013	13	JAPAN	0.01
13	2013	12	ITALY	0.19
14	2015	10	MALAYSIA	0.04
15	2018	8	NEW ZEALAND	0
16	2017	8	FRANCE	0.05
17	2016	8	SWEDEN	0.01
18	2013	8	SCOTLAND	0.02
19	2018	7	BRAZIL	0.05
20	2014	7	ROMANIA	0.01

**Table 3:** List of the top 15 keywords with the corresponding frequency.

	Count	Centrality	Year	Keywords
1	478	0.4	2003	traditional Chinese medicine
2	161	0.04	2012	systematic review
3	57	0.04	2010	Chinese medicine
4	56	0.24	2005	acupuncture
5	51	0.07	2006	herbal medicine
6	49	0.01	2011	prevalence
7	48	0.26	2004	alternative medicine
8	45	0.26	2005	complementary
9	43	0.13	2009	therapy
10	42	0.15	2012	oxidative stress
11	41	0.05	2010	artificial intelligence
12	40	0.04	2005	knowledge
13	40	0.06	2007	traditional medicine
14	37	0.01	2003	disease
15	35	0.09	2004	data mining

**Table 4:** Comparison between CM knowledge service and general medical knowledge service.

	CM Knowledge Service	General Medical Knowledge Service
Knowledge source	Static knowledge: CM-related academic journals, traditional TCM classics, and guidelines issued by professional TCM organizations. Source of case characteristic data: vision, smell, auscultation, and palpation.	Static knowledge: authoritative sources such as international medical journals, clinical guidelines, and drug registration information. Source of case characteristics data: medical examination report.
Knowledge system	CM knowledge services are mainly based on the theory and practice of CM, including CM, acupuncture, and CM diagnostics.	Based on the modern medical system, including various branches of Western medicine, such as internal medicine, surgery, pediatrics, obstetrics and gynecology, etc.
Theoretical thinking mode	Traditional Chinese medicine emphasizes syndrome differentiation and treatment, and distinguishes the etiology and pathogenesis of diseases through the four diagnostic methods of CM, such as vision, smell, auscultation, and palpation, and then chooses Chinese medicine or acupuncture and other traditional Chinese medicine treatment methods.	Focus on the physiological and pathological mechanisms of diseases, and draw up treatment plans based on large-scale clinical trials.

## Data Availability

Data are available in a publicly accessible repository. The data presented in this study are openly available in https://www.webofscience.com/wos/alldb/basic-search accessed on 10 February 2023.
